# The progressive ratio and fixed ratio 1 schedules of cocaine self-administration in rats convey the same information

**DOI:** 10.1038/s41598-022-24173-x

**Published:** 2022-11-16

**Authors:** Vladimir L. Tsibulsky, Andrew B. Norman

**Affiliations:** Department of Pharmacology and Systems Physiology, University of Cincinnati College of Medicine, Cincinnati, OH USA

**Keywords:** Neuroscience, Diseases of the nervous system, Addiction

## Abstract

Progressive ratio (PR) schedules of drug delivery are used to determine the ‘motivational’ state of an animal and drug ‘reinforcing efficacy’. This widely held interpretation is supported mainly by the observation that the PR breakpoint (BP) is proportional to the unit dose of self-administered drug. The compulsion zone theory of cocaine self-administration was applied to determine whether it can explain the pattern of lever-pressing behavior and cocaine injections under the PR schedule in rats. This theory states that cocaine induces lever pressing when levels are below the satiety threshold and above the priming/remission threshold. Rats were trained to self-administer cocaine on a fixed ratio FR1 schedule over a range of cocaine unit doses. Then they were switched to a PR schedule. Typical for the self-administration under a PR schedule, long post-injection pauses occurred when calculated cocaine levels were in the satiety zone. The compulsion zone theory interprets BP simply as the maximal number of responses which rats can perform after an injection while cocaine levels remain within the compulsion zone. The thresholds delineating the compulsion zone were very stable and independent of the self-administration schedule. PR and fixed ratio schedules convey the same pharmacokinetic/pharmacodynamic information, i.e., these two schedules are invariant.

## Introduction

Since the early days of experimentation with drug self-administration, most researchers continue to interpret this behavior within the framework of operant conditioning theory^1–7^. The basic principle of operant conditioning states that the probability of a particular behavior (lever-pressing, for example) depends upon the consequences of that behavior^8^. According to this viewpoint, many significant features of self-administration behavior may be explained only by reference to the properties of schedules of reinforcement^9^. A progressive ratio (PR) schedule requires the subject to perform an increasing number of lever presses for the next successive presentation of a reinforcer^10^. The maximal number of presses resulting in the final administration of a reinforcer was called ‘breaking point’^10^ or ‘breakpoint’ (BP). It was found that animals can press a lever many hundreds and even thousands of times between consecutive drug injections^11^. BP became a frequently used measure of relative reinforcing efficacy when different drugs, unit doses or other experimental interventions were compared^12–16^.

Conventionally, BP is a dependent measure used to quantify the degree to which the animal will work to get the next injection of the drug which serves as a reinforcer in the self-administration paradigm. Therefore, the BP magnitude should be proportional to the abuse liability of the drug and/or motivation to obtain it^12^. Detailed methodological and phenomenological analysis of drug self-administration behavior under PR schedules has been detailed previously^7,14,17^.

There is an alternative to the psychology-oriented interpretation of cocaine self-administration behavior. Cocaine is a drug and would be expected to exert its effects in vivo according to the principles of pharmacology. The compulsion zone theory assumes that drug self-administration is a drug-induced behavior^18,19^. The main goal of this study was to determine whether the compulsion zone theory of cocaine self-administration can account for all of the properties of behavior under the PR schedule of drug delivery. According to this pharmacological theory, the self-administration behavior is a succession of two drug-induced effects: (i) an initiation of responding—a priming effect; (ii) and an inhibition of the same responding—a satiety effect. Which of these two drug-induced effects occurs, at any particular time, depends on the drug concentration. There are two concentration thresholds delineating the entire range of not life-threatening cocaine concentrations into three zones. Concentrations below the minimal level which is required to induce the priming effect represent the basal or remission zone. The lower minimal level is called the priming threshold (*D*_*PT*_) when cocaine concentration rises or the remission threshold (*D*_*RT*_) for falling drug concentrations. Concentrations above the minimal level required to inhibit the priming effect represent the satiety zone. This upper threshold is called the satiety threshold (*D*_*ST*_) because animals at higher than *D*_*ST*_ levels of cocaine are in the state of satiety. Concentrations between the two thresholds represent the compulsion zone because these concentrations induce compulsive lever-pressing behavior previously during training associated with a drug injection^19^.

The compulsion zone theory suggests that a self-administration session under the fixed ratio (FR) schedule may be conveniently divided into five distinct phases. (1) *A priming phase*: the first injections of the drug initiated by an experimenter or by cue-induced lever presses before drug level reaches the priming threshold. (2) *A loading phase*: a series of dose-independent lever-presses. These injections bring cocaine levels to the satiety threshold. (3) *A maintenance phase*: a series of self-administrations under one of fixed ratio or fixed interval schedules of drug delivery. During this phase intervals between injections are dose-dependent and cocaine levels are fluctuating in quasi-steady state manner above the satiety threshold. (4) *An unloading phase*: lever presses not followed by drug injections when drug levels are exponentially falling from the satiety to the remission threshold. (5) *A remission phase*: cocaine levels remain within the remission zone (below the compulsion zone) and do not induce lever presses.

Any PR schedule, being an example of an intermittent schedule of drug delivery, may be viewed as a mixture of the two opposite schedules called continuous drug delivery (FR1) and continuous no-drug delivery (extinction schedule, according to Ferster and Skinner^20^). In other words, an injection after the last press toward the requirement defined by a PR function is followed by a series of presses without an injection. Then the cycle repeats. Therefore, the self-administration session phase under a PR schedule is a sequence of the two opposite phases of the session: loading and unloading^21,22^. The compulsion zone theory predicts that as the PR requirement increases, the ratio of remaining without consequences unloading presses to presses resulting in loading injections becomes so large that unloading prevails and the self-administration behavior inevitably ceases. Similarly, in the end of the unloading phase of FR1 self-administration sessions, the time of the last lever-press is the time when the falling drug level approaches the remission threshold (*D*_*RT*_).

This paper represents the analyses of experimental data presented at several international meetings^23–25^. Herein we present analyses of the satiety and remission thresholds during self-administration sessions under the FR1 and PR schedules of drug delivery. Results of different analyses of the same data set were presented earlier^21,22,26^.

## Results

### Baseline FR1 sessions

**Figure 1:**
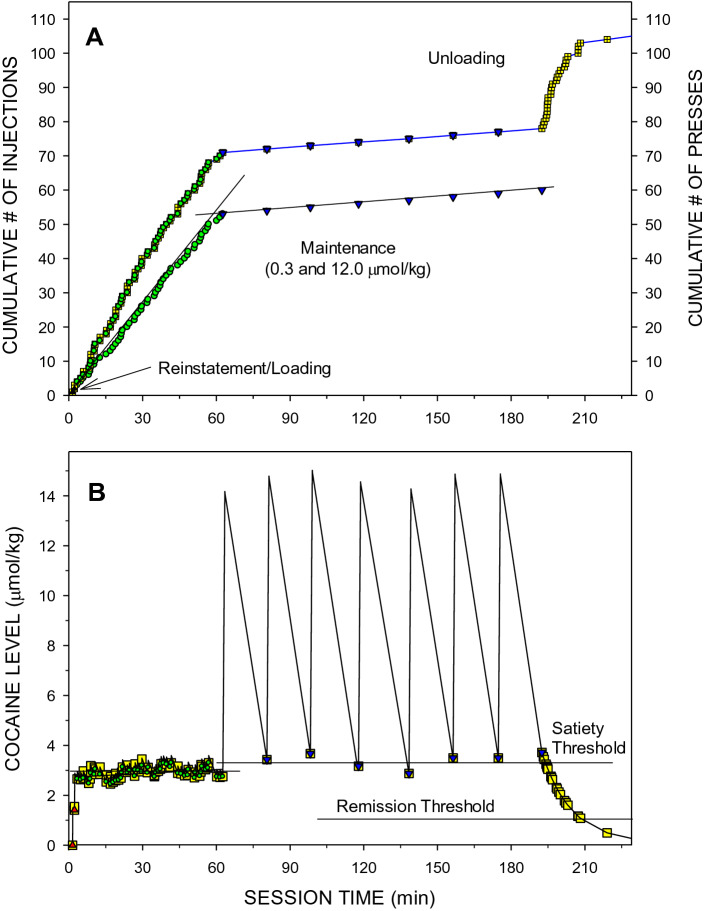
Cumulative records of events (**A**) and the calculated levels of cocaine at the time of each event (**B**) during a representative Baseline FR1 session. There were two kinds of events: lever presses (right *y*-scale) and drug injections (left *y*-scale). Red triangles represent loading injections. Yellow squares represent each active lever press. (**A**) Each symbol represents an event and the horizontal distance between each event represents an inter-event interval. The mean ± SEM inter-injection intervals were 71 ± 7 s and 1113 ± 25 s at the cocaine unit doses of 0.3 (green circle) and 12.0 (blue inverted triangle) µmol/kg, respectively. Both the inter-press and inter-injection intervals were very regular during the FR1 maintenance phase as demonstrated by the linear regression lines. The last inter press interval was 652.8 s (10.9 min), i.e. longer than the critical interval of 7.5 min^21^. The unloading phase duration was 15.7 min and the terminal local rate of lever-pressing was *LR*_*term*_ = 1.59 presses/min. (**B**) The zig-zagging line represents the calculated level of cocaine in the body at every second of the session. Horizontal lines represent mean ± SEM *D*_*ST*_ = 2.92 ± 0.03 µmol/kg and *D*_*ST*_ = 3.41 ± 0.11 µmol/kg at unit doses of 0.3 (green circle) and 12.0 (blue inverted triangle) µmol/kg, respectively, and the *D*_*RT*_ = 1.08 µmol/kg.

During the loading phase rats received 2–3 doses of 1.5 μmol/kg. After the unit dose was switched to 0.3 μmol/kg, inter-injection intervals were regular and dose dependent. Rats were more likely to press the active lever during the time-out period at low cocaine unit doses compared to high doses. That is why the slope of the cumulative number of presses is higher than the slope of the cumulative number of injections (Fig. 1A).

The estimated concentration of cocaine in the body rapidly increased during the reinstatement/loading phase and then fluctuated above the *D*_*ST*_ during the maintenance phase (Fig. 1B). Despite the huge change in the unit doses the levels of cocaine at the beginning of injections were similar. During the unloading phase cocaine levels decreased exponentially. Almost all lever presses occurred only when cocaine levels were within the compulsion zone, i.e. above the *D*_*PT*_/*D*_*RT*_ and below the *D*_*ST*_ (Fig. 1B).

### PR sessions

**Figure 2:**
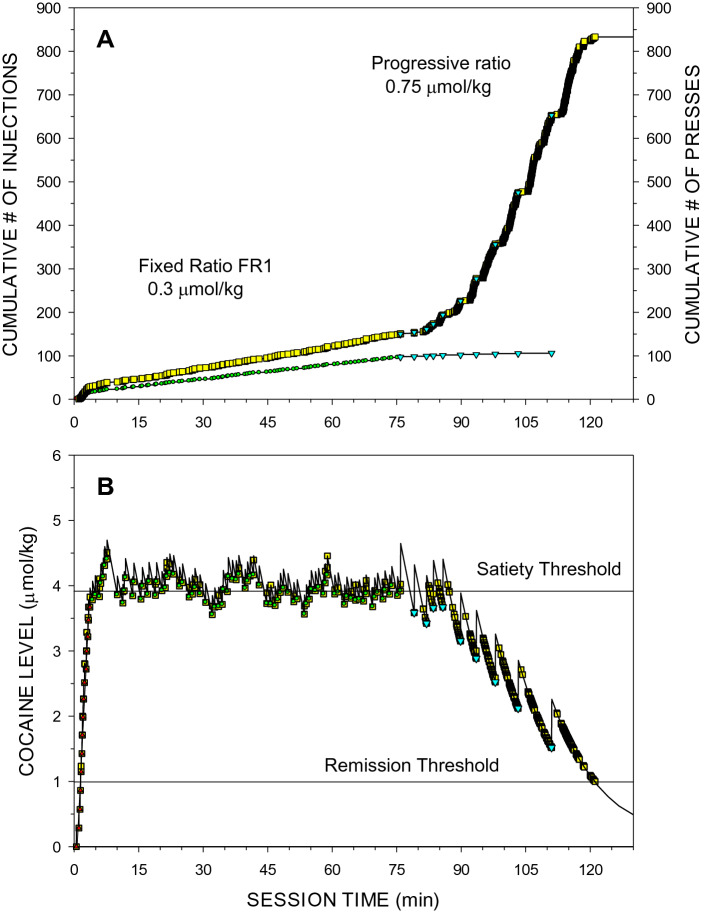
Cumulative records of events (**A**) and the calculated levels of cocaine (**B**) at the time of each event during a representative self-administration session under FR1 and PR schedules of delivery of low unit doses of cocaine (0.3 and 0.75 µmol/kg, respectively). There were two kinds of events: lever presses and drug injections. Open squares represent each active lever press. Red triangles represent loading injections. Green circles represent cocaine levels at the beginning of each injection during maintenance under the FR1 schedule at 0.3 μmol/kg unit dose. Cyan inverted triangles represent injections of 0.75 μmol/kg unit dose under the PR schedule. (**A**) The mean ± SEM inter-injection intervals during loading and maintenance phases under the FR1 schedule were 13.5 ± 2.2 s (red triangle) and 52.8 ± 3.0 s (green circle), respectively. Between post-injection pauses under the PR schedule, the mean ± SEM local inter-press interval was 3.09 ± 0.73 s (*LR* = 19.4 ± 2.7 presses/min). The last completed ratio was 178. The number of presses after the last injection but before the last press, e.g., the number of final extinction presses was 184, therefore BP = 184 presses. (**B**) Horizontal lines represent mean ± SEM *D*_*ST*_ = 3.91 ± 0.018 µmol/kg and *D*_*RT*_ = 1.00 µmol/kg.

**Figure 3:**
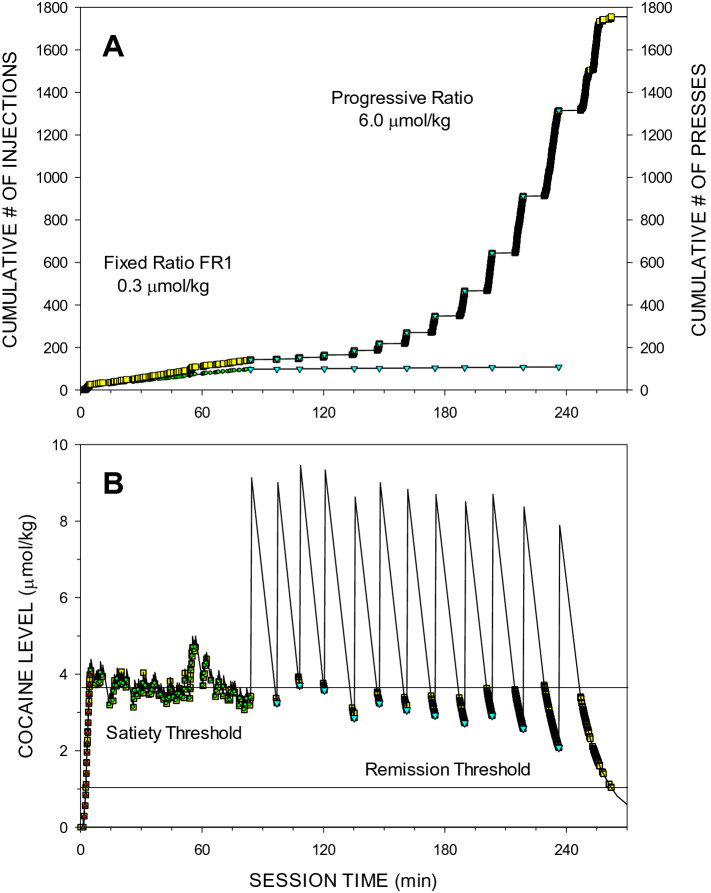
Cumulative records of events (**A**) and the calculated levels of cocaine (**B**) at the time of each event during a representative self-administration session under FR1 and PR schedules of delivery of low and high unit doses of cocaine (0.3 (green circle) and 6.0 (cyan inverted triangle) µmol/kg, respectively). For detailed figure legend see Fig. 2. (**A**) The mean ± SEM inter-injection intervals during loading and maintenance phases under the FR1 schedule were 13.1 ± 1.8 s (red triangle) and 58.9 ± 5.2 s (green circle), respectively. Between PIPs under the PR schedule, the mean ± SEM local inter-press interval was 2.53 ± 0.69 s (*LR* = 41.4 ± 6.0 presses/min). The last completed ratio was 403. The number of presses of final extinction presses was 443, therefore BP = 443 presses. (**B**) Horizontal lines represent mean ± SEM *D*_*ST*_ = 3.64 ± 0.039 µmol/kg and the *D*_*RT*_ = 1.03 µmol/kg.

Representative cumulative records of two sessions under PR schedules at two different cocaine unit doses of 0.75 and 6 μmol/kg are shown in Figs. 2 and 3. During the loading phase rats self-administered 15–20 doses of 0.3 μmol/kg at high rate. When *D*_*ST*_ was reached inter-press intervals (IPIs) abruptly increased and cocaine levels were maintained at or above the *D*_*ST*_. Note that after the 5th injection of 0.75 μmol/kg (Fig. 2) cocaine levels were below the *D*_*ST*_ all the time and post-injection pauses (PIPs) became very short. In contrast, during self-administration of 6 (Fig. 3) and 12 μmol/kg unit doses, PIPs were much longer because lever-pressing activity was suppressed while cocaine levels were above the *D*_*ST*_. The rate of presses was even higher than during the loading phase and decreased dramatically after cocaine levels fell below the *D*_*RT*_.

### Post-PR FR1 sessions

**Figure 4:**
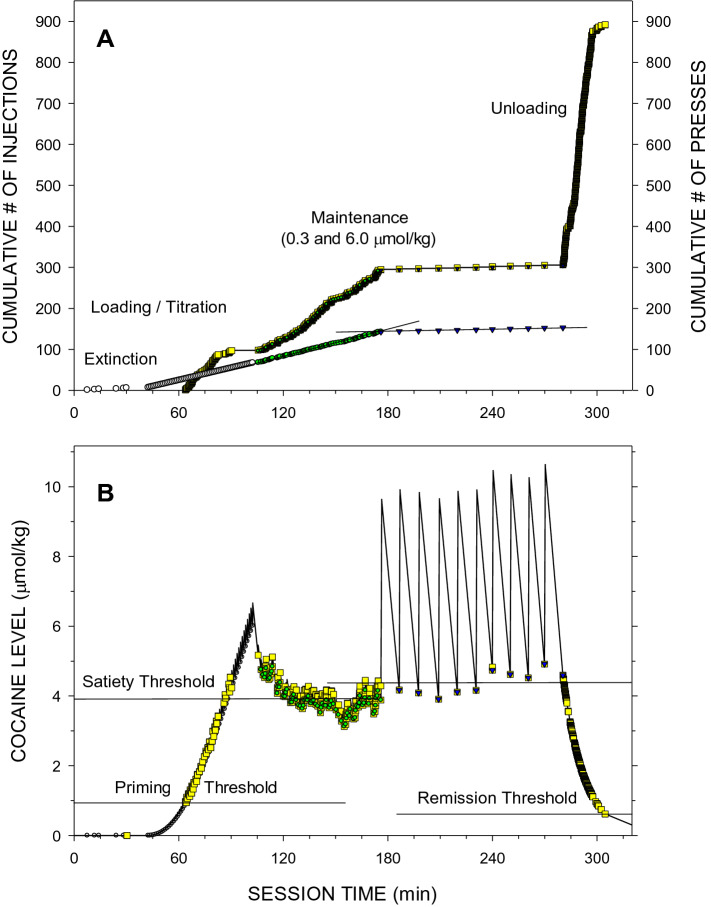
Cumulative records of events (**A**) and the calculated levels of cocaine at the time of each event (**B**) during a representative Post-PR FR1 session. Lines and symbols as in Fig. 1. (**A**) Open circles represent programmed priming injections of escalating doses of cocaine (doses increased from zero to 0.655 μmol/kg). Both the inter-press and inter-injection intervals were very regular during the FR1 maintenance phase as demonstrated by the linear regression lines. The mean ± SEM inter-injection intervals were 56.4 ± 4.5 s and 626.2 ± 26.0 s at unit doses of 0.3 µmol/kg (green circle) and 6 µmol/kg (blue inverted triangle), respectively. The last inter press interval was longer than 60 min. The unloading phase duration was 24.1 min and the terminal local rate of lever-pressing was *LR*_*term*_ = 24.27 presses/min. (**B**) Horizontal lines represent mean ± SEM *D*_*ST*_ = 3.89 ± 0.04 µmol/kg and *D*_*ST*_ = 4.38 ± 0.10 µmol/kg at unit doses of 0.3 (green circle) and 6.0 (blue inverted triangle) µmol/kg, respectively, and the* D*_PT _= 0.97 µmol/kg and the *D*_*RT*_ = 0.62 µmol/kg.

Rats placed into the experimental chambers typically displayed some cue-induced lever-pressing activity which quickly extinguished (Fig. 4). After a 30 min period without any presses, non-contingent loading injections were initiated (Fig. 4A) in order to titrate the *D*_*PT*_. The titration continued until cocaine levels reached 6.5 µmol/kg that is higher than the *D*_*ST*_ when lever-pressing activity was inhibited (Fig. 4B). Then cocaine levels exponentially decreased back to the *D*_*ST*_ and lever presses resumed. The maintenance phase under the FR1 schedule started with the cocaine dose of 0.3 µmol/kg and continued with different cocaine unit doses. During the unloading phase cocaine level decreased exponentially and the lever-pressing activity was the highest of all session phases until the cocaine level fell below the *D*_*RT*_. The probability of time-out presses to occur, especially at the lower unit dose was significantly higher than during the Baseline FR1 sessions (Fig. 4). Furthermore, the total number of unloading presses was dramatically higher in the Post-PR FR1 sessions (Fig. 4) than during the Baseline FR1 sessions (Fig. 1).

### Comparison of D_ST_ between sessions of different kinds

The two independent methods assessing *D*_*ST*_ were used. Both methods are based on the assumption of the one-compartment pharmacokinetic (PK) model and both require knowledge of cocaine doses and inter-injection intervals. The first method, based on the satiety threshold model of maintained cocaine self-administration (Eq. 2), estimates both the *D*_*ST*_ and *t*_1/2_ values. Because this method requires knowledge of dose-duration function at the wide range of unit doses, it could be applied only to the Baseline FR1 and Post-PR FR1 sessions. The second method was based on the assumption of a cocaine elimination rate constant value that may be applied in real time during any self-administration session. The satiety thresholds estimated by the first method and calculated by the second method are shown in Table 1.

**Table 1:** Estimated and calculated cocaine *D*_*ST*_ and estimated cocaine elimination half-life *t*_1/2_ in individual rats.

Rat	Baseline FR1 sessions			PR sessions		Post-PR FR1 sessions		
	Estimated^a^ *D*_*ST*_ (µmol/kg)	Estimated *t*_1/2_ (s)	Calculated^b^ *D*_*ST*_ (µmol/kg)	Mean^c^ *t*_1/2_ (s)	Calculated *D*_*ST*_ (µmol/kg)	Estimated *D*_*ST*_ (µmol/kg)	Estimated *t*_1/2_ (s)	Calculated *D*_*ST*_ (µmol/kg)
1	3.86 ± 0.49^d^	479 ± 44	4.07 ± 0.19	482	3.67 ± 0.08	3.86 ± 0.71	485 ± 61	3.71 ± 0.16
2	4.12 ± 0.96	462 ± 85	3.82 ± 0.46	529	4.45 ± 0.12	5.23 ± 1.03	596 ± 81	5.52 ± 0.34
3	4.05 ± 0.34	489 ± 29	4.39 ± 0.09	523	3.64 ± 0.07	4.29 ± 0.42	556 ± 37	3.85 ± 0.10
4	5.15 ± 0.51	451 ± 33	4.62 ± 0.16	453	3.43 ± 0.16	4.93 ± 0.21	454 ± 12	3.52 ± 0.57
5	4.77 ± 0.45	559 ± 38	5.70 ± 0.13	585	4.37 ± 0.13	5.39 ± 1.09	611 ± 84	4.93 ± 0.19
6	4.61 ± 0.14	468 ± 11	3.86 ± 0.53	510	4.81 ± 0.16	5.29 ± 0.97	552 ± 73	5.20 ± 0.20
Mean	4.43 ± 0.20	485 ± 16	4.41 ± 0.29	514 ± 18	4.06 ± 0.23	4.82 ± 0.25	542 ± 25	4.45 ± 0.35

^a^Estimated using Eq. (2). ^b^Calculated using the estimated half-lives. ^c^Mean of Baseline FR1 and Post-PR FR1 sessions. ^d^Mean ± SEM.

All three parameters, estimated *D*_*ST*_ and *t*_1/2_ and calculated *D*_*ST*_, showed significant individual variability (Kruskal–Wallis ANOVA on Ranks, *p* = 0.002). Estimated *D*_*ST*_ did not differ between the Baseline FR1 and Post-PR FR1 sessions (paired *t* = 2.004, *p* = 0.1). Estimated cocaine half-lives were statistically different before and after the PR phase of the experiment (paired *t* = 2.833, *p* = 0.037). Therefore, the mean *t*_1/2_ values of Baseline FR1 and Post-PR FR1 sessions were used for PR sessions. Values for *D*_*ST*_ were calculated during FR1 sessions at the beginning of every injection assuming 500 s cocaine half-life and then were corrected using means of estimated cocaine half-lives in individual rats (Table 1). Calculated *D*_*ST*_ did not differ from estimated *D*_*ST*_ within each type of the session and also between Baseline FR1 sessions, PR sessions and Post-PR FR1 sessions.

### Comparison of D_RT_ between the different types of sessions

Both *D*_*RT*_ and *D*_*PT*_ during the session were calculated according to estimated half-lives in individual rats (Table 2). Calculated *D*_*RT*_ and *D*_*PT*_ showed significant individual variability (Kruskal–Wallis ANOVA on Ranks, *p* = 0.002). The calculated *D*_*RT*_ did not change significantly between Baseline FR1, PR or Post-PR FR1 sessions (paired *t*-tests). There was no significant difference between *D*_*PT*_ calculated in the beginning of Post-PR FR1 sessions and *D*_*RT*_ calculated in the end of either Baseline FR1, PR or Post-PR FR1 sessions.

**Table 2:** Calculated *D*_*RT*_ and *D*_*PT*_ in individual rats.

Rat	Baseline FR1 *D*_*RT*_ (µmol/kg)	PR sessions *D*_*RT*_ (µmol/kg)	Post-PR FR1 *D*_*RT*_ (µmol/kg)	Post-PR FR1 *D*_*PT*_ (µmol/kg)
1	0.95 ± 0.13^a^	0.95 ± 0.08	0.91 ± 0.11	1.84 ± 0.14
2	2.13 ± 0.24	1.05 ± 0.01	1.36 ± 0.07	2.52 ± 0.14
3	1.19 ± 0.12	1.14 ± 0.11	1.05 ± 0.12	0.77 ± 0.14
4	0.99 ± 0.08	0.40 ± 0.05	0.38 ± 0.05	0.71 ± 0.14
5	2.01 ± 0.12	1.79 ± 0.07	1.96 ± 0.26	2.07 ± 0.14
6	1.62 ± 0.41	0.98 ± 0.09	1.66 ± 0.23	2.05 ± 0.14
Mean	1.48 ± 0.21	1.05 ± 0.18	1.22 ± 0.23	1.66 ± 0.30

^a^Mean ± SEM.

### Comparison of compulsion zone width and unloading presses between sessions

Compulsion zone width was calculated as *D*_*ST*_–*D*_*RT*_ (Table 3). Calculated compulsion zone widths showed significant individual variability (Kruskal–Wallis ANOVA on Ranks, *p* = 0.002). There were no significant differences between Baseline FR1, PR or Post-PR FR1 sessions (paired *t*-tests). Linear regression analysis showed a significant correlation between the calculated *D*_*ST*_ and *D*_*RT*_ in Baseline FR1 sessions (*F*_1,152_ = 9.28, *r* = 0.24, *p* = 0.003). This correlation strengthened after PR training and remained strong during Post-PR FR1 sessions (*F*_1,138_ = 12.44, *r* = 0.29, *p* < 0.001 and *F*_1,59_ = 13.44, *r* = 0.43, *p* < 0.001, respectively).

**Table 3:** Calculated compulsion zone width in individual rats.

Rat №	Baseline FR1 *D*_*ST*_–*D*_*RT*_ (µmol/kg)	PR sessions *D*_*ST*_–*D*_*RT*_ (µmol/kg)	Post-PR FR1 *D*_*ST*_–*D*_*RT*_ (µmol/kg)
1	3.72 ± 0.25^a^	2.72 ± 0.12	2.83 ± 0.18
2	3.54 ± 0.42	3.35 ± 0.15	4.33 ± 0.26
3	3.73 ± 0.22	2.52 ± 0.13	2.78 ± 0.18
4	3.97 ± 0.18	3.04 ± 0.16	3.91 ± 1.00
5	3.69 ± 0.88	2.57 ± 0.14	3.08 ± 0.13
6	3.07 ± 0.88	3.83 ± 0.15	3.74 ± 0.30
Mean	3.62 ± 0.12	3.00 ± 0.21	3.45 ± 0.26

^a^Mean ± SEM.

### Comparison BP at the plateau and unloading presses

There was no significant difference between BP at the plateau of the dose-BP function (BP at 6 and 12 µmol/kg cocaine doses combined, Table 4) and the total number of unloading presses during Post-PR FR1 sessions after self-administration of the same doses (paired *t* = 0.698, *p* = 0.516).

**Table 4:** BP at two highest cocaine unit doses and the number of unloading presses.

Rat №	PR sessions Mean BP at 6–12 µmol/kg	Post-PR FR1 Unloading presses
1	412 ± 52^a^	432 ± 35
2	267 ± 64	263 ± 36
3	334 ± 67	455 ± 131
4	477 ± 75	276 ± 126
5	97 ± 14	57 ± 46
6	594 ± 111	517 ± 149
Mean	364 ± 71	333 ± 69

^a^Mean ± SEM.

## Discussion

### Correlation between lever-pressing activity and cocaine level

The cocaine satiety threshold and half-life were estimated according to the satiety threshold model of maintained cocaine self-administration^28,29^. Cocaine levels were calculated every second during self-administration sessions according to one-compartment PK model and assuming that the average half-life of cocaine was 500 s^31^. Calculated cocaine levels at the satiety and remission thresholds were recalculated (post hoc correction) using estimated half-lives in individual rats. These two different methods produced two very similar values for the satiety threshold with the averaged difference within 5% accuracy. This result suggests that we can assume with a high degree of confidence that recalculated values for *D*_*ST*_*, D*_*RT*_ and the width of the compulsion zone were also accurate.

The compulsion zone theory of cocaine self-administration states that (i) cocaine levels below the priming/remission thresholds do not induce lever presses, (ii) cocaine levels between the priming and satiety threshold induce stereotypic lever presses, and (iii) cocaine levels above the satiety threshold inhibit the same stereotypic presses^19^. Visual analysis of Panels B in Figs. 1, 2, 3 and 4 representing both lever presses and calculated cocaine levels during all three types of sessions is consistent with these theoretical statements.

### Long term constancy of D_ST_ and D_RT_ but not t_1/2_

Training under the PR schedule resulted in a dramatic 12-fold increase in lever-pressing activity^22^. In this study we demonstrated that both the satiety and remission thresholds remained constant during and after PR training, i.e. both thresholds are activity-rate independent. In spite of a substantial variability of these thresholds between animals there was a significant within animals correlation between *D*_*ST*_ and *D*_*RT*_ suggesting that both thresholds may be under control of the same regulatory mechanism. This correlation was stronger after PR training when the rate of lever presses was much higher allowing more precise measurement of the *D*_*RT*_.

The pharmacodynamic (PD) parameters (*D*_*ST*_ and *D*_*RT*_) demonstrated stability over the entire duration of this experiment, longer than 5 months. At the same time the pharmacokinetic (PK) parameter (*t*_1/2_) slightly but significantly increased over this time. This finding is consistent with a reported slowdown of pharmacokinetics in aging organisms^32^. Cocaine concentrations in brain and blood was higher in adult compared to peri adolescent mice after acute or chronic i.p. administration of 20 mg/kg of cocaine^33^.

### Behavior under the PR schedule explained by PK/PD mechanisms

In all sessions, the lever-pressing activity substantially decreased when the cocaine level approached the remission threshold and almost completely ceased when cocaine levels were too low to induce lever presses^22^. In PR sessions, at the time of cocaine level crossing the remission threshold, the rate of lever presses dramatically slowed down and the next ratio requirement could not be reached. We hypothesize that the mechanism of BP is the same as the mechanism of termination of unloading phase after FR1 self-administration. If so, the unloading presses might be viewed as PR self-administration at zero cocaine dose. It explains why BP values at very low unit doses are at the level of 30–40% of the maximal BP values observed at the highest unit doses^21,34^. The observation that the cocaine level at BP and termination of the unloading phase after FR1 self-administration are the same is consistent with their parameters being mediated by the same PK/PD mechanism.

The dose-BP function has two phases^21,35^. Initially BP increased with the dose of cocaine. However, at cocaine doses slightly higher than 3 μmol/kg (~ 1 mg/kg) the function reached a plateau. This is because at doses higher than the width of the compulsion zone (Table 3), the peak cocaine levels after every injection should be always above the satiety threshold. Therefore, the ratio requirements reached the maximal value when cocaine level transited across the entire compulsion zone resulting in the same number of lever presses regardless of the unit dose exceeding the width of the compulsion zone. It is also consistent with the finding that there was no significant difference between BP at the plateau of dose-BP function and the total number of unloading presses after self-administration in Post-PR FR1 sessions. This is because in both cases the rate of lever presses and the width of the compulsion zone were the same (Table 4).

Comparison of Figs. 2 and 3 helps to explain why intervals between injections increased and post-injection pauses gradually decreased from the first to the last injection. The higher ratio requirements needed more time to accomplish, and this resulted in longer intervals between injections. Longer intervals resulted in cocaine levels falling further below the satiety threshold at the time of completion of the requirement for the next injection. Therefore, at a particular unit dose there was a shorter time when the cocaine levels were above the satiety threshold, the major contributor to the post-injection pauses. It remains unexplained why PIPs, although brief, were still observed even when the cocaine levels were always below the *D*_*ST*_ at low cocaine unit dose.

The duration of cocaine levels staying within the compulsion zone increased with progression of ratios but also as a function of the cocaine unit dose. Longer durations resulted in higher BP values as a function of the dose. Therefore, dose-BP function represents a dose-duration function^26^ and not a dose-magnitude of response function as it was previously assumed^12^.

## Conclusions

Self-administration under the FR1 schedule at different cocaine unit doses provides information about the key PK/PD and behavioral parameters defining cocaine self-administration: the priming, satiety and remission thresholds, the drug elimination half-life, and the rate of compulsive lever-pressing. Training under the PR schedule does not affect the first four parameters but, as we have shown previously, dramatically increases the rate of cocaine-induced lever presses (the behavioral parameter). According to the compulsion zone theory of cocaine self-administration, BP indicates the time when cocaine levels fall to the *D*_*RT*_ below which cocaine-induced lever presses cease. It also explains other features of PR behavior: the shape of the dose-BP function, the dose-dependent increase in post-injection pauses and the gradual increase in inter-injection intervals.

## Materials and methods

### Cocaine self-administration training

Six male Sprague–Dawley rats (from Harlan, Indianapolis, IN, initial body weight 180–200 g and 400–500 g over the duration of the studies) were housed individually on a 12-h light–dark cycle (lights on at 6:00 a.m.) and food and water were available ad lib. All studies were conducted in accordance with the National Institutes of Health *Guide for the Care and Use of Laboratory Animals* and under a protocol approved by the Institutional Animal Care and Use Committee at the University of Cincinnati and reported in accordance with ARRIVE guidelines. There is a limitation of this research: only male rats were included. Further studies are needed to determine sex differences.

Rats were surgically implanted with an indwelling catheter into the right jugular vein under halothane anesthesia^27^. Beginning 14 days after the surgery, rats were trained to self-administer cocaine HCl using the FR1 schedule with a time-out period equal to injection durations but not less than 5 s (TO5-40 s). Training at the unit dose of 1.5 μmol/kg (0.51 mg/kg) cocaine HCl continued until individual rats met the criterion of stable maintained self-administration. This criterion was no significant change of the mean and standard deviation of the inter-injection intervals between five consecutive sessions. At the same time the proportion of inactive lever presses was lower than 2.5% of the total presses. The duration of access to cocaine during these training sessions did not exceed three hours.

The chambers, hardware, materials and procedures of maintaining catheter patency were described in detail previously^21^. The concentration of the self-administered cocaine solution was 40 μmol/ml. The unit doses ranged from 0.3 to 12 μmol/kg and were regulated by the duration of the injection, which ranged approximately from 2 to 40 s. In order to quickly reach the *D*_*ST*_ during the initial loading phase of a session, the first unit doses of cocaine were programmed to be 1.5 μmol/kg. After the self-administration of the initial loading doses, the program switched subsequent doses to the preset maintenance dose in random order between sessions.

### Experimental design

The design of this experiment was also published previously^22^. Here is the brief description. There were three kinds of self-administration sessions. Rats placed into the experimental chambers typically displayed some cue-induced lever-pressing activity. These first presses initiated cocaine priming injections. During Baseline FR1 sessions rats received two doses of cocaine. The first unit dose of cocaine was selected from 0.3, 0.75 and 1.5 µmol/kg and the second unit dose was selected from the range of 3.0, 6.0 and 12.0 µmol/kg. The next series of PR sessions started exactly like the baseline FR1 sessions with 0.3 µmol/kg under the FR1 schedule followed by self-administration of one of unit doses under the PR schedule. The unit dose for the PR phase of the session was randomly selected from the following: 0.75, 1.5, 3, 6 or 12 μmol/kg. Progressive function was

1 $${\text{Ratio }} = {\text{ truncate }}\left( {{5}\cdot\left( {{\text{exp}}\left( {j\cdot\left( {n{-}{ 1}} \right)} \right) \, {-}{ 1}} \right)} \right) \, + { 1,}$$

where the slope factor *j* = 0.4 generated ratios: 1, 3, 7, 12, 20, 32, 51, 78, 118, 178, 268, 403, 603, 902, 1348, 2013 (see details in Refs.^21,22,26^).

The last series of sessions (Post-PR FR1 sessions) started with the *D*_*PT*_ measurements. In order to distinguish a cue-induced priming from a cocaine-induced priming, rats were placed into the experimental chambers, but syringe pumps were disconnected. Lever presses turned light signals on, but rats did not receive any injections. The extinction was completed after 30 min without any presses. Then *D*_*PT*_ was titrated by programmed injections of escalating doses of cocaine until rats resumed lever presses induced now by cocaine (for more detailed description of the priming method see Ref.^21^). After the last programmed priming injection, the session continued exactly as in Baseline FR1 sessions. A summary of the experimental timeline is presented in a Supplementary Information file.

### Cocaine satiety threshold calculation

The *D*_*ST*_ and cocaine half-life values were calculated for each rat using non-linear regression analysis of the mean inter-injection intervals during the maintenance phase of each session as a function of the unit dose according to the equation:

2 $$T = {\text{ ln}}\left( {{1 } + D_{U} /D_{ST} } \right)\cdot t_{{{1}/{2}}} /{\text{ln(2),}}$$

where *T* is the mean inter-injection interval, *D*_*U*_ is the unit dose, *D*_*ST*_ is the satiety threshold and *t*_1/2_ is the elimination half-life of the drug^28,29^. The mean ± SEM of the values from the individual rats was then calculated for the group of six rats. The estimates of three thresholds (priming, satiety, and remission) were compared between Baseline FR1 sessions, PR sessions and Post-PR FR1 sessions. The priming threshold (*D*_*PT*_) was defined as in Ref.^30^, the satiety threshold as in Refs.^28,29^ and the remission threshold as in Refs.^21,22^.

### Cocaine level estimations

Cocaine levels in the body of individual rats were computed every second of the session according to one compartment model and assuming cocaine elimination half-life of 500 s (for detailed method see Ref.^31^).

### Priming threshold measurement

During the session designed for the *D*_*PT*_ measurement (Post-PR FR1 sessions), rats were placed in individual cages and the time of each lever press was recorded. Initial attempts to self-administer cocaine, presumably induced by environmental cues, interfered with the measurement of the cocaine-induced priming effect. Therefore, non-contingent presentations of the signal light above the lever were given at random intervals of several minutes. Thirty minutes after the last lever press the catheter was automatically filled with the cocaine solution and non-contingent injections of escalating doses of cocaine were administered every minute until the peak cocaine level reached 6.5 µmol/kg. This limit was selected on the basis of previous experiments because it was clearly above the *D*_*ST*_. Reinstatement of cocaine self-administration was defined as a series of lever presses following the first 5 lever presses made with inter-press intervals shorter than 2 min. Once lever pressing was reinstated, the *D*_*PT*_ was calculated as the mean of peak cumulative cocaine levels after the ultimate and penultimate priming injections preceding the first 5 reinstatement presses.

### Statistical analyses

All statistical and visual analyses of data were conducted using SigmaPlot software v. 14.0 (Systat Software, Palo Alto, CA, https://systatsoftware.com). The distinct phases of the self-administration session were differentiated on the basis of abrupt and sustained changes in the inter-press or inter-injection intervals. The transition from the unloading to remission phase was determined using method described in Ref.^22^. Presses between the end of the post-injection pause and the next injection, and also presses between the first and last unloading press were used to calculate the terminal local rate (*LR*_*term*_) of lever-pressing activity. Only the inter-injection intervals during the maintenance phase under the FR1 schedule of each session were used to calculate a mean value and for further regression analysis of the dose-interval function. The dose-interval relationship for each rat was estimated on the basis of at least five sessions per each cocaine unit dose. The *D*_*ST*_ and *t*_1/2_ for the group were estimated as the mean ± SEM of the values from the individual rats (n = 6). Paired *t*-test, Pearson correlation and a two-way ANOVA were used to test for a significant difference with the significance criterion set at *α* = 0.05.

## Supplementary Information

Supplementary Figure 1.

## Data Availability

The datasets used and/or analyzed during the current study are available from the corresponding author on reasonable request. These data sets are extensive and require the program SigmaPlot to examine. Please refer to data files for Rats 387, 390, 393, 395, 397, and 400, which completed all of the phases of this study.
