# Supplementary material for: The progressive ratio and fixed ratio 1 schedules of cocaine self-administration in rats convey the same information
Source: Sci Rep. 2022 Nov 16;12:19683. doi: 10.1038/s41598-022-24173-x (PMC9668967; doi:10.1038/s41598-022-24173-x)
Supplement: Supplementary file 1 — Supplementary Figure 1. [file 41598_2022_24173_MOESM1_ESM.docx]

Supplementary Material.

Fig. 1. The timeline of the experimental procedures. The surgical implantation of the first jugular catheter was on Day 0. On Day 14 ± 0 (mean ± SEM, n = 6 rats) after the recovery period, self-administration sessions started and continued 5 days a week. On Day 23.2 ± 1.1, rats acquired stable lever-pressing behavior. On Day 40.5 ± 0.5, rats met the criterion of stable maintained self-administration and inter-injection intervals at different cocaine unit doses were determined during the Baseline FR1 sessions. On Day 109.5 ± 1.9, the schedule was switched to the PR. Between Day 162 ± 0.4 and Day 195 ± 5.5, the drug delivery schedule alternated between FR1 and PR. The experiment continued on the FR1 schedule until Day 223.5 ± 6.5.
